# Protein kinase C-α signals P115RhoGEF phosphorylation and RhoA activation in TNF-α-induced mouse brain microvascular endothelial cell barrier dysfunction

**DOI:** 10.1186/1742-2094-8-28

**Published:** 2011-04-08

**Authors:** Jing Peng, Fang He, Ciliu Zhang, Xiaolu Deng, Fei Yin

**Affiliations:** 1 Department of Pediatrics, Xiangya Hospital of Central South University, No.87 Xiangya Road, Changsha, Hunan, 410008, China

## Abstract

**Background:**

Tumor necrosis factor-**α **(TNF-**α**), a proinflammatory cytokine, is capable of activating the small GTPase RhoA, which in turn contributes to endothelial barrier dysfunction. However, the underlying signaling mechanisms remained undefined. Therefore, we aimed to determine the role of protein kinase C (PKC) isozymes in the mechanism of RhoA activation and in signaling TNF-**α**-induced mouse brain microvascular endothelial cell (BMEC) barrier dysfunction.

**Methods:**

Bend.3 cells, an immortalized mouse brain endothelial cell line, were exposed to TNF-**α **(10 ng/mL). RhoA activity was assessed by pull down assay. PKC-**α **activity was measured using enzyme assasy. BMEC barrier function was measured by transendothelial electrical resistance (TER). p115RhoGEF phosphorylation was detected by autoradiography followed by western blotting. F-actin organization was observed by rhodamine-phalloidin staining. Both pharmacological inhibitors and knockdown approaches were employed to investigate the role of PKC and p115RhoGEF in TNF-**α**-induced RhoA activation and BMEC permeability.

**Results:**

We observed that TNF-**α **induces a rapid phosphorylation of p115RhoGEF, activation of PKC and RhoA in BMECs. Inhibition of conventional PKC by Gö6976 mitigated the TNF-**α**-induced p115RhoGEF phosphorylation and RhoA activation. Subsequently, we found that these events are regulated by PKC-**α **rather than PKC-β by using shRNA. In addition, P115-shRNA and n19RhoA (dominant negative mutant of RhoA) transfections had no effect on mediating TNF-**α**-induced PKC-**α **activation. These data suggest that PKC-**α **but not PKC-β acts as an upstream regulator of p115RhoGEF phosphorylation and RhoA activation in response to TNF-**α**. Moreover, depletion of PKC-**α**, of p115RhoGEF, and inhibition of RhoA activation also prevented TNF-**α**-induced stress fiber formation and a decrease in TER.

**Conclusions:**

Taken together, our results show that PKC-**α **phosphorylation of p115RhoGEF mediates TNF-**α **signaling to RhoA, and that this plays a critical role in signaling F-actin rearrangement and barrier dysfunction in BMECs.

## Background

The integrity of brain microvascular endothelial cells (BMECs) is the basis of the maintenance of the central nervous system (CNS) microenvironment [1]. Tumor necrosis factor-**α **(TNF-**α**) is released in large amounts by macrophages, monocytes and other leukocytes in response to gram-positive or gram-negative bacterial substances, and plays a vital role in the pathogenesis of infectious brain edema [2]. RhoA has been implicated in signaling by TNF-**α**, lysophosphatidic acid (LPA), and hepatocyte growth factor (HGF), and is known to play a critical role in regulating endothelial barrier function [3]. We previously demonstrated that elevated TNF-**α **is highly correlated with the occurrence of blood brain barrier (BBB) dysfunction, and that inhibiting Rho kinase by pretreatment with Y-27632 alleviates brain edema in animals after TNF-**α **challenge [4]. Thus, these findings suggest an essential role for the RhoA/Rho kinase pathway in the regulation of TNF-**α**-induced BMECs barrier dysfunction. However, little is known about the complex signaling events regulating RhoA, by which TNF-**α **plays a role in BMEC barrier dysfunction.

RhoA serves as a molecular switch, cycling between active GTP-bound and inactive GDP-bound states regulated by a large number of activators and inactivators, including guanine nucleotide exchange factors (GEFs), GTPase-activating proteins (GAPs), and guanine nucleotide dissociation inhibitors (GDIs). Activation of RhoA requires GDP/GTP exchange, which is controlled by GEFs. More than 80 RhoGEFs have been identified, with the characteristic of containing a Dbl homology (DH) domain as well as a pleckstrin homology (PH) domain [5]. Among several GEFs identified, p115RhoGEF can directly link heterotrimeric Gα12/13 subunits to RhoA regulation [6,7]. It has been shown that thrombin binds to and cleaves protease-activated receptor (PAR-1) in endothelial cells, leading to activation of heterotrimeric G-protein Gq1, Gi, and Gα12/13 [8]. Several studies have shown that the p115RhoGEF/RhoA pathway is required for several stimuli such as thromboxane A2 and LPA-induced endothelial barrier leakage [9,10]. Thus, these studies suggest that TNF-**α **induces BMEC hyperpermeability, possibly through a p115RhoGEF/RhoA-dependent mechanism.

Protein kinase C isozymes are serine-hreonine kinases that phosphorylate multiple proteins, which in turn regulate intracellular signaling. We previously showed in vitro that LPA-induced BBB breakdown was associated with activation of PKC and was prevented by the PKC inhibitor Ro31-8220 by down-regulating the claudin-5 expression and F-actin recombination [11]. Several studies have demonstrated a convergence between PKC and the RhoA pathway in regulating endothelial barrier dysfunction [12]. PKC-**α **and RhoA coimmunoprecipitate in the particulate fraction of colon smooth muscle cells in response to different contactile agonists [13]. A recent study suggests that PKC-**α **can trigger RhoA activation and promote actin cytoskeletal changes in thrombin-induced endothelial cell hyperpermeability [14]. It is assumed that PKC signaling is involved in RhoA activation and subsequently endothelial barrier breakdown. Taken together, these data suggested the possibility that PKC and p115RhoGEF work together in RhoA activation and endothelial barrier dysfunction. However, there are no studies on how PKC and p115RhoGEF signaling interact in the pathogenesis of TNF-**α**-induced RhoA activation and barrier dysfunction in BMECs.

Here we took advantage of both pharmacological inhibitors and knockdown approaches to investigate the role of PKC and p115RhoGEF in TNF-**α**-induced RhoA activation and BMEC permeability. Our data show that PKC-**α **but not PKC-β mediates p115RhoGEF phosphorylation, which in turn triggers RhoA activation, and then promotes F-actin rearrangement and barrier permeability in BMECs in response to TNF-**α**.

## Methods

### Reagents

Anti-p115RhoGEF, PKC-**α **and PKC-β were purchased from Santa Cruz Biotechnology (San Diego, CA, USA). HRP-linked anti-goat and -rabbit IgG, and RhoA antibodies, were purchased from Cell Signaling (Beverly, MA, USA). A RhoA pull-down kit containing GST-RhoAtekin-RhoA-binding domain beads was purchased from Cytoskeleton (Denver, CO, USA). TNF-**α **was obtained from Sigma Chemical (St. Louis, MO, USA). Gö6976 was purchased from Calbiochem (Gibbstown, NJ, USA). Fibronectin-coated cell inserts with 0.4 μm pore size were obtained from BD Biosciences (Franklin Lakes, NJ, USA). Lipofectamine 2000 and rhodamine-phalloidin were purchased from Invitrogen (Carlsbad, CA, USA).

### Cell culture

Bend.3 cells, mouse brain-deprived microvascular endothelial cells, were kindly afforded by Dr. Zhang Jian (The University of Chicago, Chicago, USA) and were cultured in Dulbecco's modified Eagle's medium (DMEM) containing 10% (v/v) fetal bovine serum at 37℃ 5% CO_2_. Culture medium was changed every 2 days. All experiments were performed in confluent monolayers on day 9 or 10 post seeding.

### Plasmids and transfection

PcDNA3.1hygro-n19RhoA plasmid, the dominant negative mutant of RhoA, was synthesized in Minghong CO (CHN). This mutant was obtained by in vitro site-directed mutagenesis of Thr to Asn at codon 19, which maintains RhoA in an inactive GDP-loaded state. An expression vector containing PcDNA3.1hygro plasmid alone served as the control of the PcDNA3.1hygro-n19RhoA plasmid.

PLKO.1-puro-PKC**α**-shRNA and PLKO.1-puro-PKCβ-shRNA were gifts from Dr. Zhang Jian (The University of Chicago, Chicago, USA). P115RhoGEF-shRNA was purchased from Shanghai GeneChem Co (CHN), and was constructed into the PLKO.1-puro expression vector. An empty PLKO.1-puro vector was used as the control for the PKC**α**-shRNA and p115RhoGEF-shRNA plasmids.

All of the plasmids were introduced into Bend.3 cells by using Lipofectamine 2000 according to the manufacturer's instructions. The stable transfected Bend.3/n19RhoA (n19RhoA) and Bend.3/PcDNA3.1hygro cells (vector-1) were obtained by using the Hygromycin B (400 ug/ml, Sigma) selection method after transfection. The Bend.3 cells transfected with PLKO.1-puro-PKC**α**-shRNA, PLKO.1-puro-PKCβ-shRNA, PLKO.1-puro-p115RhoGEF-shRNA and empty PLKO.1-puro plasmids were called PKC**α**-shRNA, PKCβ-shRNA, p115-shRNA and vector-2 cells respectively. All of them were used for experiments after selection by Puromycin (300 ug/ml). The inhibition levels of RhoA activity and PKC-**α **expression as well as p115RhoGEF were detected by pull-down assay and western blot respectively.

### Assay of activated RhoA

RhoA activity was measured using a RhoA pull-down kit according to the manufacturer's protocols. Briefly, subconfluent cell cultures were starved with serum-free medium for 6 hours, then stimulated by TNF-**α **before an ice-cold PBS rinse and lysis in 500 μl of the supplied lysis buffer. Equal volumes of supernatants were incubated with RhoAtekin-RBD affinity beads for 1 hour at 4°C, followed by two washes in lysis buffer and three washes in the supplied wash buffer. Bound proteins were eluted in 5 × 1% SDS sample buffer and examined by 12% SDS-PAGE and western blot with anti-RhoA antibody analysis. Aliquots of total lysate were also analyzed for the amount of RhoA present.

### PKC-α kinase activity assay

PKC-**α **activity was measured using PKC-**α **Assay Kits. Cell cultures were starved with serum free medium for 6 hours then stimulated by TNF-**α **before an ice-cold PBS rinse and lysis in 500 μl of NP-40 lysis buffer. The cell lysates were then incubated in PKC-**α **antibody (2 ug antibody/800 ug protein) with a rotor shaker, at 4°C overnight. Then 20 ul ProteinA/G agarose were added into the cell lysates, which were then centrifuged and washed with PBS 5 times. After a final aspiration, 5 uL of KRREILSRRPSYR substrate, 5 uL of the ATP solution, and 15 ul PKC kinase buffer were added to initiate the kinase reaction which was carried out at 30-35°C for 60 min with constant shaking. The kinase reaction was stopped with 20 uL 2 × SDS-sample loading buffer and boiling for 2 min. Results were then examined using 12% SDS-PAGE and western blot with anti-KRREILSRRPpSYR antibody analysis.

### Measurement of transendothelial electrical resistance (TER)

Endothelial permeability was assayed by measuring TER using a Millicell ERS Voltohmmeter (Millipore, Billerica, MA, USA), and the values are shown as Ω•cm2 based on culture inserts. The TER of cell-free inserts was subtracted from the TER of filters with cells. The TER of cells was measured before and after treatment with TNF-**α**.

### Actin staining

Cell monolayers were stained with Rhodamine-phalloidin to examine the structure of filamentous (F)-actin. Washed cells were fixed with polyoxymethylene, washed again, and permeabilized for 5 min with 0.1% Triton X-100. The cells were incubated with a 1% solution of BSA (30 min, RT), and stained with Rhodamine-phalloidin (0.20 mol/L, 30 min, RT, in dark conditions). Stained F-actin was visualized using an OLYMPUS XB-51 fluorescence inverted microscope under 200-fold magnification.

### Immunoblot analysis

Protein samples (30 μg total protein per lane) were subjected to 8% or 12% SDS-PAGE, and the proteins were then electrophoretically transferred to a polyvinylidene fluoride (PVDF) membrane blocked by 5% BSA for 1 h at room temperature and then incubated with antibodies overnight at 4°C. Secondary antibody was incubated for 1 h at room temperature. A chemiluminescence reagent, ECL western blotting detection reagent (Amersham Biosciences Corp., Arlington Heights, IL, USA), was used to make the labeled protein bands visible. The blots were developed by the enhanced chemiluminescence method (Amersham).

### Phosphorylation of p115RhoGEF

After serum deprivation for 6 h, BMECs were labeled with 150 uCi/ml ^32^P for 4 h in phosphate-free MEM. Cells were then stimulated with TNF-**α **(10 ng/ml) for the indicated times, quickly transferred onto ice, washed with ice-cold PBS containing 500 uM Na3VO4 and lysed. After centrifugation, the cleared lysate was incubated with either control IgG or anti-mouse P115RhoGEF Ab for 2 h followed by the addition of protein A/G plus agarose beads overnight. The beads were then collected by centrifugation, washed with detergent-free buffer and 2 ug/mL each of pepstatin A, leupeptin, and aprotinin. The above procedures were performed at 4°C. Protein from each sample was eluted by boiling the beads in SDS sample buffer, electrophoresing on 8% SDS-polyacrylamide gels, and transfer to nitrocellulose for visualization of p115RhoGEF phosphorylation by autoradiography, followed by western blotting with p115RhoGEF antibody to verify equal protein loading. Specificity of the p115RhoGEF antibody was confirmed using normal mouse IgG as a negative control.

### Statistical analyses

All of the data are expressed as the means ± SD. A Student's *t*-test was performed to determine the significant difference between two groups. One-way ANOVA analysis followed by Student-Neuman-Keuls post-hoc tests was utilized to determine the significant differences among multiple groups. P < 0.05 was considered to be statistically significant.

## Results

### TNF-α activates RhoA, mediating barrier dysfunction in Bend.3 cells

**Figure 1:**
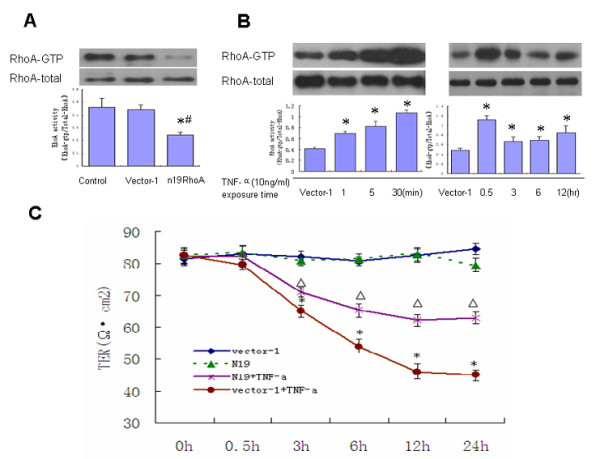
**TNF-α activates RhoA, which mediates barrier dysfunction in Bend.3 cells**. **(A) **PcDNA3.1hygro-n19RhoA (the dominant negative mutant of RhoA), and PcDNA3.1hygro vector plasmids (vector-1) were transfected into Bend.3 cells by Lipofectamine 2000. Stably transfected cells were obtained using the Hygromycin B (400 ug/ml) selection method after transfection. Untreated Bend.3 cells served as controls. The inhibitory effect of n19RhoA was confirmed by pull-down assay. **(B) **Levels of active RhoA in vector-1 cells after TNF-**α **(10 ng/ml) treatment were detected at various time points by pull-down assay to detect relative amounts of RhoA-GTP. Total RhoA levels served as loading controls. The results show that TNF-**α **induced a rapid activation of RhoA within 1 min, reaching peak activation within 30 min. (n = 3 independent experiments). **(C) **Vector-1- and n19RhoA-transfected cells were treated with or without TNF-**α **for various time periods. TER assay was used to determine Bend.3 monolayer permeability. The results show that TNF-**α**-induced vector-1 cell barrier breakdown was partially alleviated in n19RhoA cells. *: p < 0.05 vs. Vector-1, #: p < 0.05 vs. Control, △: p < 0.05 vs. Vector-1+TNF-a n = 4/group.

To address the direct involvement of RhoA in TNF-**α**-induced Bend.3 cell barrier permeability, n19RhoA cells (dominant negative mutant of RhoA) were used to suppress activation of RhoA. The remarkable inhibitory effect of n19RhoA was confirmed by pull-down assay (Figure 1A).

TNF-**α **(10 ng/ml) exposure induced rapid and prolonged RhoA activation in a time-course manner. RhoA activity increased significantly at 1 min, with a maximum response occurring at 30 min followed by a decline at 3 h. However, RhoA activity remained higher than the baseline even 12 h after TNF-**α **administration (Figure 1B).

Measurements of transendothelial electrical resistance (TER) reflecting endothelial monolayer permeability changes showed that administration of TNF-**α **(10 ng/ml) resulted in a time-dependent decrease in TER. The TER of vector-1 cells and n19RhoA cells without TNF-**α **challenge remained stable enough to be regarded as the baseline. Compared with the baseline, the TER of vector-1 cells with TNF-**α **dropped to the lowest level at 12 h. However, inhibiting RhoA activity with n19RhoA cells significantly suppressed decreases in TER in response to TNF-**α **(Figure 1C). These data indicate that TNF-**α **activate RhoA, which mediates barrier dysfunction in Bend.3 cells.

### TNF-α-induced RhoA activation is secondary to PKCα activation

**Figure 2:**
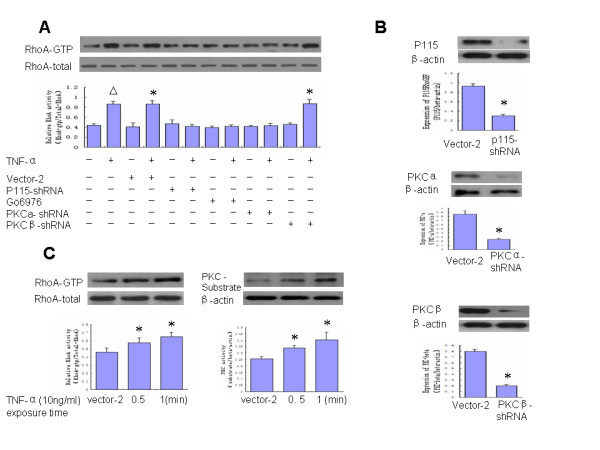
**Protein kinase C-α and p115RhoGEF regulate TNF-α-induced RhoA activation**. **(A) **Bend.3 cells were pretreated with or without Gö6976 (1 μM), a selective inhibitor of conventional PKC isozymes. RhoA activation was assayed after 5 min of TNF-**α **challenge. The results show that Gö6976 pretreatment of Bend.3 cells blocked TNF-**α**-induced RhoA activation. Bend.3 cells were transfected with vector-2(empty PLKO.1-puro vector), PKC**α**-shRNA, PKCβ-shRNA or p115-shRNA in the presence or absence of TNF-**α**. RhoA activation was partially prevented in PKC**α**-shRNA and P115-shRNA transfected cells, whereas RhoA activation did not affect PKCβ-shRNA transfected cells. **(B) **The Bend.3 cells transfected with vector-2, PKC**α**-shRNA, PKCβ-shRNA and p115-shRNA respectively. Stably transfected cells were used for experiments after selection by Puromycin (300 ug/ml). The inhibitory levels of p115RhoGEF (upper), PKC-**α **(middle) and PKC-β (bottom) expression were confirmed by western blot. **(C) **The time course of PKC-**α **and RhoA activation after TNF-**α **(10 ng/ml) treatment was detected. The results showed that TNF-**α**-induces rapid activation of PKC-**α **as well as RhoA at 0.5 min. (n = 3 for each condition). *: p < 0.05 vs. Vector-2, △: p < 0.05 vs. untreated Bend.3 cells. -, absence; +, presence.

To address the question of whether PKC acts upstream of RhoA activation, Gö6976, a selective inhibitor of conventional PKC isoenzymes, was used to inhibit the activity of PKC-**α **and PKC-β. Gö6976 pretreatment of Bend.3 cells blocked TNF-**α**-induced RhoA activation, implicating conventional PKC as an upstream regulator of RhoA activation (Figure 2A).

To identify the specific conventional PKC isozymes regulating the activation of RhoA, PKC**α**-ShRNA and PKCβ-ShRNA were used. The significant knockdown effect of PKC**α**-ShRNA (Figure 2B, middle) and PKCβ-shRNA (Figure 2B, bottom) was confirmed by western blot. As shown in Figure 2A, depletion of PKC-β failed to abrogate RhoA activation in response to TNF-**α **in Bend.3 cells, while knockdown PKC-**α **significantly blocked RhoA activation. These data provide unequivocal evidence that PKC-**α **but not PKC-β is critical in stimulating TNF-**α**-induced RhoA activation.

To further confirm if PKC-**α **is the upstream regulator of RhoA, the time course of PKC-**α **and RhoA activation was compared, and the effects of n19RhoA transfection on PKC**α **activation were assessed. Although TNF-**α **induced rapid activation of PKC-**α **as well as RhoA at the same time (Figure 2C), n19RhoA expression had no effect on mediating changes of PKC-**α **activity in Bend.3 cells (Figure Four B). This finding indicates that PKC-**α **signaling acts as an upstream regulator in TNF-**α**-induced RhoA activation in Bend.3 cells.

### TNF-α-induced RhoA activation is secondary to p115RhoGEF phosphorylation

To address the question of whether p115RhoGEF phosphorylation is also involved in TNF-**α**-induced RhoA activation, P115-shRNA was used to deplete p115RhoGEF expression. The remarkable knockdown effect of P115-shRNA was confirmed by western blot (Figure 2B, upper).

**Figure 3:**
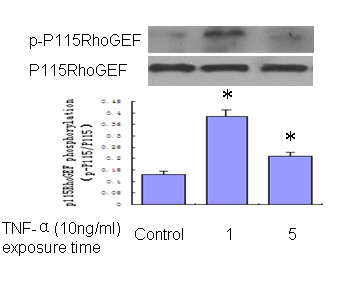
**TNF-α induces P115RhoGEF phosphorylation in Bend.3 cells**. Serum-starved cells were labeled with ^32^P for 4 h in phosphate-free medium. Bend.3 cells were then stimulated with TNF-**α **for the indicated times and p115RhoGEF phosphorylation was detected by autoradiography followed by western blotting. Untreated Bend.3 cells served as controls. TNF-**α **induced rapid p115RhoGEF phosphorylation within 1 min. (n = 3 independent experiments). *P < 0.05 vs. Control. -, absence; +, presence.

Figure 3A shows the autoradiograph of p115RhoGEF phosphorylation in ^32^P. The results show that TNF-**α **induced a surprisingly fast p115RhoGEF phosphorylation reaching maximum at 1 min (Figure 3). P115-shRNA transfected cells prevented TNF-**α**-induced RhoA activation, implicating p115RhoGEF as one of the upstream regulators of RhoA activation in response to TNF-**α **(Figure 2A).

### PKC-α but not PKC-β activation is the upstream signal in TNF-α-induced p115RhoGEF phosphorylation

**Figure 4:**
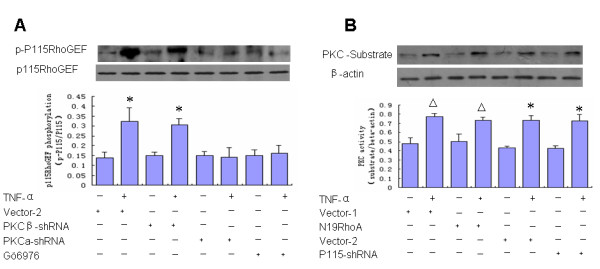
**PKC-α but not PKC-**β **is the upstream signal in TNF-α-induced P115RhoGEF phosphorylation**. **(A) **Serum-starved cells were transfected with vector-2, PKC**α**-shRNA, PKCβ-shRNA or pretreated with Gö6976. Serum-starved cells were then labeled with ^32^P for 4 h in phosphate-free medium. Cells were stimulated with TNF-**α **for 1 min, and then p115RhoGEF phosphorylation was detected by autoradiography followed by western blotting. The results show that depletion of PKC-**α **by Gö6976 or PKC**α**-ShRNA prevents the phosphorylation of p115RhoGEF in response to TNF-**α**. In contrast, depletion of PKC-β isozyme expression by using PKCβ-shRNA had no effect on P115RhoGEF phosphorylation. **(B) **Serum-starved cells were transfected with vector-1, n19RhoA, vector-2 and P115-shRNA. Cells were stimulated with TNF-**α **for 1 min, and then PKC-**α **activation was detected by vitro enzyme assasy. The results show that n19RhoA and P115-shRNA transfections had no effect on PKC-**α **activation. (n = 3 for each condition). *: p < 0.05 vs. Vector-2, △: p < 0.05 vs. Vector-1.-, absence; +, presence.

An attempt was made to explore if PKC-**α **is the upstream signal for TNF-**α**-mediated p115RhoGEF phosphorylation in BMECs. We found that depletion of PKC-**α **by Gö6976 or PKC**α**-ShRNA prevented the phosphorylation of p115RhoGEF in response to TNF-**α**, whereas depletion of PKC-β by PKCβ-ShRNA had no effect on p115RhoGEF phosphorylation (Figure 4A). Our experiment further demonstrated that P115-shRNA transfection attenuates p115RhoGEF expression, but has no effect on PKC-**α **activation (Figure 4B). These data suggest that PKC-**α **but not PKC-β acts as an upstream regulator of p115RhoGEF phosphorylation in TNF-**α **challenge.

### Role of the PKC-α/p115RhoGEF/RhoA pathway in TNF-α-induced F-actin rearrangement and BMEC barrier dysfunction

**Figure 5:**
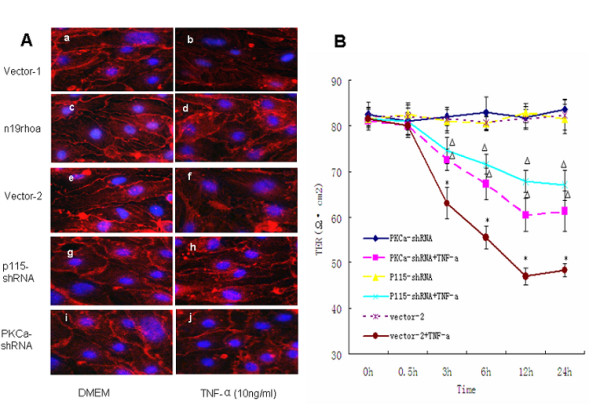
**Role of the PKC-α/P115RhoGEF/RhoA pathway in TNF-α-induced F-actin rearrangement and BMEC barrier dysfunction**. **(A) **F-actin remodeling and paracellular gap formation were detected by immunofluorescence. Serum-starved cells were transfected with vector-1, n19RhoA, vector-2, P115-shRNA and PKCa-shRNA for 3 h with either DMEM (left) or TNF-**α **(right). Before stimulation, Bend.3 cells did not display stress fibers although they exhibited an extensive cortical actin network (Figure 5A.a,e). After 3 h of TNF-**α **exposure, cells exhibited prominent stress fiber formation and paracellular gaps (Figure 5A.b,f), which were partially prevented by inhibition of RhoA activation (Figure 5c-d), and depletion of p115RhoGEF (Figure 5g-h) as well as PKC-**α **expression (Figure 5 i-j). (n = 3 independent experiments). **(B) **Vector-2, P115-shRNA and PKCa-shRNA transfected cells were treated with or without TNF-**α **for various time periods. Bend.3 monolayer permeability was determined by TER. The results show that knockdown of p115RhoGEF and PKC-**α **can alleviate TNF-**α**-induced barrier breakdown. *: p < 0.05 vs. vector-2, △: p < 0.05 vs. vector-2 +TNF-a, n = 4/group.

We analyzed the effect of RhoA inactivation, P115RhoGEF and PKC-**α **knockdown on TNF-**α**-induced F-actin dynamics by immunofluorescence (Figure 5A) and barrier permeability by TER (Figure 5B). Before stimulation, Bend.3 cells did not display stress fibers although they exhibited an extensive cortical actin network (Figure 5A.a,e). After 3 h of TNF-**α **exposure, cells exhibited prominent stress fiber formation and paracellular gaps (Figure 5A.b,f). However, the stress fiber formation and intra-cellular gaps induced by TNF-**α **were reduced by inhibiting the activation of RhoA (Figure 5A.c-d), p115RhoGEF (Figure 5A. g-h) and PKC-**α **(Figure 5A. i-j). Furthermore, as shown from Figure 5 B, after exposure to TNF-**α **for 12 h, the TER of cells with p115RhoGEF depletion and PKC-**α **displayed as 67.8 ± 2.49 and 60.5 ± 3.64 Ω•cm^2^, higher than that of vector-2 cells (47 ± 1.87 Ω•cm^2^). This indicates inhibition of RhoA activation (Figure 1D), and suggests that depletion of p115RhoGEF and PKC-**α **could alleviate TNF-**α**-induced barrier breakdown. Moreover, according to our data, the inhibitor of p115RhoGEF acted more efficiently than the inhibitor of PKC-**α **in repairing the TER.

## Discussion

BMECs, which are linked by tight junctions, act as a physical and metabolic barrier to shield the brain from toxic substances in the blood, supply brain tissues with nutrients, and filter harmful compounds from the brain back into the bloodstream [15]. However, the normal physiological functions of the endothelium are perturbed during serious sepsis. It has been shown that TNF-**α **contributes to endothelial barrier breakdown and cytokine transport across the blood-brain barrier (BBB) in sepsis [16]. Direct i.v. injection of recombinant TNF-**α **also can induce BBB opening [17]. Therefore, identification of the inflammatory signaling initiated by TNF-**α **in BMECs is paramount to understanding the mechanisms of infectious brain edema.

RhoA is a key regulator of cytoskeletal dynamics, actin stress fiber formation, and myosin phosphorylation, and thus by inference, in the control of endothelial barrier function [18,19]. However, the involvement of RhoA signaling has recently been challenged. Opposing studies have indicated that Y-27632 does not counteract either histamine-induced microvascular leakage in the airway [20] or an LPS-induced increase in permeability of skeletal muscle [21]. It is possible that different cell types and different stimuli regulate different signal transduction pathways for altering endothelial cell permeability. In our study, TNF-**α **(10 ng/mL) induces a robust activation of RhoA from 1 min up to 12 h (Figure 1B). The TER of Bend.3 and vector-1 with TNF-**α **decreased at 30 min, and dropped to the lowest level at 12 h. However, down-regulation of RhoA activation by n19RhoA, a dominant negative mutant of RhoA, remarkably suppressed TER decrease in response to TNF-**α **treatment (Figure 1C). These data suggest that RhoA participates in TNF-**α**-induced mouse BMEC barrier dysfunction. However, the mechanisms of the activation of RhoA, and thereby the loss of endothelial barrier integrity, have not been elucidated.

As RhoGEF, a family of guanine nucleotide exchange factors, provides a direct pathway for regulation of RhoA, in this study we addressed the basis of RhoA activation and its contribution in mediating the loss of endothelial barrier function induced by TNF-**α**. RhoGEF catalyses the exchange of GDP for GTP by promoting an active conformation of the small monomeric GTPase RhoA, which enables the recruitment of effector proteins that mediate downstream effects[22-24]. As a direct link between Gα12/13 and RhoA, recruitment of p115RhoGEF to the plasma membrane has been observed in response to LPA and thromboxane A2 [25,26]. It has been reported that activation of the serum response factor (SRF) is not only dependent on Gα12/13-linked GPCRs and RhoA, but also on over-expression of p115RhoGEF [27]. It is possible that TNF-**α **activates RhoA, resulting in up-regulation of p115RhoGEF. Our data also show that TNF-**α **induces rapid phosphorylation of p115RhoGEF in Bend.3 cells that could be detected at 1 min (Figure 3). Depletion of p115RhoGEF in Bend.3 cells greatly impaired RhoA activation (Figure 2A) and attenuated BMEC barrier dysfunction (Figure 1C) in response to TNF-**α**, indicating a critical role for P115RhoGEF in TNF-**α**-associated RhoA activation.

Aside from Gα12/13 directly stimulating the exchange activity of p115RhoGEF on RhoA, there may be additional regulatory pathways contributing to p115RhoGEF phosphorylation. Our previous study showed that PKC-**α **is expressed in primary cultured BMECs and astrocytes. Inhibition of PKC attenuates LPA-induced BBB permeability [11]. How PKC alters endothelial permeability remains an interesting question. Several studies have suggested that the endothelial contractile response could be triggered by a PKC-dependent activation of the RhoA pathway [28-30]. TNF-α induces a time-dependent increase in PKC mRNA expression and RhoA activation in vascular tissues [31]. More specifically, conventional PKC phosphorylates Rho-GDP dissociation inhibitor (GDI) on serine 34, resulting in a specific decrease in affinity for RhoA, leading to nucleotide exchange and interaction with downstream effectors [32]. Furthermore, PKC is a phospholipid-dependent serine/threonine kinase involved in diverse intracellular signal transduction processes [33]. Since p115RhoGEF contains a sequence for phosphorylation, we addressed the possibility that PKC may mediate RhoA activation by inducing the phosphorylation of p115RhoGEF.

Our results provide several lines of evidence that p115RhoGEF phosphorylation and RhoA activation are mediated by a PKC-dependent pathway in BMECs. We show that TNF-**α**-induced p115RhoGEF phosphorylation occurs concurrently with TNF-**α**-induced activation of RhoA. Furthermore, inhibition of PKC by Gö6976, a specific conventional isozyme-selective inhibitor of PKC, abrogated not only TNF-**α**-induced RhoA activation but also p115RhoGEF phosphorylation. Subsequently, we narrowed this effect specifically to PKC-**α **by using both pharmacological inhibitors and knockdown approaches. Our results reveal that treatment of BMECs with PKCβ-shRNA fails to prevent RhoA activation and p115RhoGEF phosphorylation in response to TNF-**α**. However, knockdown of PKC-**α **by PKC**α**-ShRNA successfully blocked marked RhoA activation and p115RhoGEF phosphorylation. In addition, P115-shRNA and n19RhoA transfection had no effect on mediating TNF-**α**-induced PKC-**α **activation. Taken together, these results indicate that PKC-**α **is critical in regulating TNF-**α**-induced p115RhoGEF phosphorylation and RhoA activation in BMECs.

BMEC permeability is precisely controlled by cell contact protein complexes (focal adhesions, adherens junctions and tight junctions) and cytoskeletal elements (F-actin, microtubules, and intermediate filaments) [34,35]. F-actin plays an important role in maintaining the integrity of the tight junction complex, and therefore in modulating the permeability of the BBB [36,37]. Reduction of TER and rearrangement of F-actin are good indicators of barrier dysfunction. Here we detected them to observe the functional relevance of PKC-α/p115RhoGEF/RhoA pathway in signaling endothelial barrier disruption. The results show that TNF-**α **causes a significant decrease in TER in BMECs transfected with vector-2 alone (Figure 5B). However, this response was significantly reduced in cells transfected with n19RhoA (Figure 1D), p115-shRNA or PKC**α**-shRNA (Figure 5B). These effects were accompanied by decreases in the amount of stress fibers and paracellular gaps (Figure 5A). Thus, these results indicate that the PKC-**α**/p115RhoGEF/RhoA pathway is the mechanism mediating TNF-**α**-induced dynamics of F-actin and elevation of BMEC permeability, which in turn might contribute to infectious brain edema.

## Conclusion

Collectively, our data provide the first direct evidence that PKC-**α **phosphorylation of p115RhoGEF is involved in TNF-**α**-induced RhoA activation, and that this plays a critical role in signaling increased barrier permeability in mouse brain endothelial cells.

## Competing interests

The authors declare that they have no competing interests.

## Authors' contributions

JP carried out the study and data analyses, and drafted the manuscript. FH participated in the molecular biology studies, and made modifications to the paper. CLZ participated in the cell culture and plasmid transfection. XLD carried out the immunofluorescence, and performed TER measurement. FY participated in the conceptualization and design of the study. All authors edited and approved the final manuscript.
